# Activation of Nrf2/HO-1 Pathway by Nardochinoid C Inhibits Inflammation and Oxidative Stress in Lipopolysaccharide-Stimulated Macrophages

**DOI:** 10.3389/fphar.2018.00911

**Published:** 2018-09-04

**Authors:** Jin-Fang Luo, Xiu-Yu Shen, Chon Kit Lio, Yi Dai, Chun-Song Cheng, Jian-Xin Liu, Yun-Da Yao, Yang Yu, Ying Xie, Pei Luo, Xin-Sheng Yao, Zhong-Qiu Liu, Hua Zhou

**Affiliations:** ^1^Faculty of Chinese Medicine, Macau University of Science and Technology, Macau, China; ^2^State Key Laboratory of Quality Research in Chinese Medicines, Macau University of Science and Technology, Macau, China; ^3^Institute of Traditional Chinese Medicine and Natural Products, College of Pharmacy, Jinan University, Guangzhou, China; ^4^College of Pharmacy, Hunan University of Chinese Medicine, Changsha, China; ^5^Joint Laboratory for Translational Cancer Research of Chinese Medicine of the Ministry of Education of the People’s Republic of China, Guangzhou University of Chinese Medicine, Guangzhou, China

**Keywords:** *Nardostachys chinensis*, Nardochinoid C, Nrf2, HO-1, ROS

## Abstract

The roots and rhizomes of *Nardostachys chinensis* have neuroprotection and cardiovascular protection effects. However, the specific mechanism of *N. chinensis* is not yet clear. Nardochinoid C (DC) is a new compound with new skeleton isolated from *N. chinensis* and this study for the first time explored the anti-inflammatory and anti-oxidant effect of DC. The results showed that DC significantly reduced the release of nitric oxide (NO) and prostaglandin E_2_ (PGE_2_) in lipopolysaccharide (LPS)-activated RAW264.7 cells. The expression of pro-inflammatory proteins including inducible nitric oxide synthase (iNOS) and cyclooxygenase-2 (COX-2) were also obviously inhibited by DC in LPS-activated RAW264.7 cells. Besides, the production of interleukin-6 (IL-6) and tumor necrosis factor-α (TNF-α) were also remarkably inhibited by DC in LPS-activated RAW264.7 cells. DC also suppressed inflammation indicators including COX-2, PGE_2_, TNF-α, and IL-6 in LPS-stimulated THP-1 macrophages. Furthermore, DC inhibited the macrophage M1 phenotype and the production of reactive oxygen species (ROS) in LPS-activated RAW264.7 cells. Mechanism studies showed that DC mainly activated nuclear factor erythroid 2-related factor 2 (Nrf2) signaling pathway, increased the level of anti-oxidant protein heme oxygenase-1 (HO-1) and thus produced the anti-inflammatory and anti-oxidant effects, which were abolished by Nrf2 siRNA and HO-1 inhibitor. These findings suggested that DC could be a new Nrf2 activator for the treatment and prevention of diseases related to inflammation and oxidative stress.

## Introduction

Multiple inflammatory diseases, including RA (McInnes and Schett, 2011), AD (Akiyama et al., 2000), seriously endanger human health. It is well accepted that inflammation is linked with oxidative stress. Oxidative stress refers to elevated intracellular levels of ROS that is considered to be the most potent inflammatory mediators (Shin et al., 2008). Antioxidants play a significant role in reducing inflammation (Waxman, 1996). The activation of nuclear factor erythroid 2-related factor 2 (Nrf2) pathway could inhibit the progression of inflammation (Xu et al., 2003). Nrf2-mediated antioxidant gene expression can reduce the macrophage M1 phenotype and ROS production (Kobayashi et al., 2016). Since Nrf2 pathway plays a critical role in inflammation, Nrf2 activators has become a potential therapeutic strategy for numerous disorders (Crunkhorn, 2012), such as inflammatory disorders (Kim et al., 2010), cardiovascular diseases (Li et al., 2009), neurodegenerative diseases (Joshi and Johnson, 2012), cancer (Sporn and Liby, 2012), type 2 diabetes (Chartoumpekis and Kensler, 2013), chronic kidney disease (Ruiz et al., 2013) and multiple sclerosis (Gold et al., 2012). However, there are only very little Nrf2 activators in clinics. Tecfidera (dimethyl fumarate), a potent Nrf2 activator, has been approved for the treatment of multiple sclerosis (Gold et al., 2012), but long-term use of this drug can cause resistance and other side effects (Deeks, 2014). So, the discovery of new and safer Nrf2 activator for clinical use has become an important task in drug discovery.

In recent years, the research showed that natural components extracted from plant have anti-inflammatory and antioxidant effects (Shen et al., 2014). The roots and rhizomes of *Nardostachys chinensis* have been used for blood disorders, herpes and infection (Arora, 1965; Pakrashi and Chatarji, 1994), the extracts of *N. chinensis* were also used for the treatment of epilepsy and hysteria (Bagchi et al., 1991). Above all, *N. chinensis* have neuroprotection and cardiovascular protection properties. However, the action mechanism of *N. chinensis* remains unclear. There are some studies reporting that the compounds isolated from *N. chinensis* suppressed LPS-induced activation of RAW264.7 cells (Hwang et al., 2012; Shin et al., 2015). The activation of Nrf2-mediated antioxidant pathway has the neuroprotective effect (Catino et al., 2016) and antioxidant could promote anti-inflammatory effect (Li et al., 2008).

**FIGURE 1:**
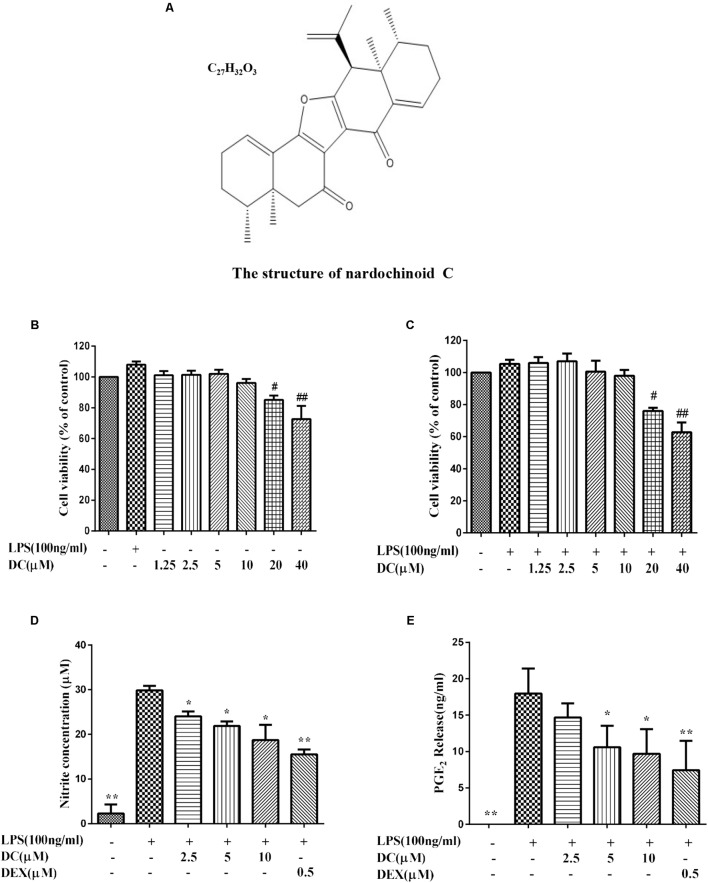
The effect of Nardochinoid C (DC) on the release of NO and PGE_2_ in LPS-stimulated RAW264.7 cells. **(A)** The chemical structure of DC. **(B)** Cytotoxicity of DC on LPS-unstimulated RAW264.7 cells. **(C)** Cytotoxicity of DC on LPS-stimulated RAW264.7 cells. The cells were treated with DC at various concentrations (1.25–40 μM) for 1 h, and then stimulated with or without LPS for 18 h, the cell viability was analyzed with MTT method. Effects of DC on the productions of NO **(D)** and PGE_2_ **(E)** in LPS-stimulated RAW264.7 cells. The cells were incubated with indicated concentrations of DC or DEX for 1 h, and then stimulated with LPS for 18 h. The concentration of NO (expressed as nitrite) and PGE_2_ in the culture medium were quantified by ELISA kits. Statistical analysis was carried out by using one-way ANOVA with Tukey’s multiple comparison tests in GraphPad Prism7 (*P* < 0.05, ANOVA). Results are expressed as mean ± SEM of three independent experiments (*N* = 3), ^#^*p* < 0.05, ^##^*p* < 0.01, vs. LPS-unstimulated cells **(B,C)** or ^∗^*p* < 0.05, ^∗∗^*p* < 0.01, vs. LPS-stimulated cells **(D,E)**.

Until now, the antioxidant activity of the compounds extracted from *N. chinensis* in macrophages remain unknown. Therefore, the anti-inflammatory activity and the antioxidant effect of Nardochinoid C (DC) (**Figure 1A**), a new compound with new skeleton isolated from *N. chinensi*s was studied for the first time in this research.

Macrophages play a key role in the innate immune response. It serves as the first line of defense in the body against invading pathogens and promotes cell protection and repair processes (Linde et al., 2007). Activated macrophage produces a variety of pro-inflammatory mediators, such as interleukin -6 (IL-6), tumor necrosis factor-α (TNF-α), prostaglandin E_2_ (PGE_2_), and nitric oxide (NO) (Noguchi et al., 2003; Kang et al., 2006; Szekanecz and Koch, 2007), which can promote the development of inflammatory (Coussens and Werb, 2002).

Therefore, two inflammatory cell models, LPS-stimulated RAW264.7 macrophage and LPS-stimulated THP-1 macrophage, were chosen to examine the anti-inflammatory activity of DC in this study.

We found that: (1) DC had significant anti-inflammatory activity both in LPS-induced RAW264.7 cells model and LPS-induced THP-1 cells model. (2) DC produced anti-inflammatory effect mainly through activating Nrf2/HO-1 pathway, rather than inhibiting NF-κB and MAPK pathways in LPS-stimulated RAW264.7 cell. (3) DC activated Nrf2 antioxidant pathways to reduce ROS production in LPS-stimulated RAW264.7 cell. (4) DC produced anti-inflammatory effect mainly through increasing the expression and the activity of HO-1 antioxidant protein.

These findings suggest that DC could be a new potential Nrf2 activator for the treatment and prevention of diseases related to inflammation and oxidative stress.

## Materials and Methods

### Materials

DC (HPLC purity > 98%) was obtained from the Institute of Traditional Chinese Medicine and Natural Products, Jinan University. LPS, SFN, DEX, ZnPP, PMA, hemin, bilirubin, NADPH, glucose-6-phosphate, glucose-6-phosphate dehydrogenase and antibody to α-Tubulin were obtained from Sigma (St. Louis, MO, United States). LPS was first dissolved in PBS and then diluted with the medium to get the final working concentration. All the other test compounds (DEX, SFN, DC, ZnPP, and hemin) were first dissolved in DMSO and then diluted with the medium or potassium phosphate buffer to reach the final working concentration, respectively. The final concentration of DMSO was less than 0.1%. Antibodies to iNOS, COX-2, p-IKKα/β, p-p65, IKKα/β, p65, p-JNK, p-ERK, p-p38, JNK, ERK, p38, Nrf2, and Keap1 were from Cell Signaling Technology (Boston, MA, United States). Antibodies to p62, HO-1 and NQO-1 were from Abcam (Abcam, Cambridge, United Kingdom). Griess reagent from Promega (Promega, United States). ELISA kit for PGE_2_ were from Cayman Chemical (Cayman Chemical, Ann Arbor, MI, United States), ELISA kits for IL-6 and TNF-α were from eBioscience (eBioscience, Inc., United States). siRNA for Nrf2 (sc-37049), non-specific siRNA (sc-37007) and antibody to β-actin were from Santa Cruz Biotechnology (Santa Cruz, CA, United States). Lipofectamine 2000 (Lipo2000) was obtained from Invitrogen (Carlsbad, CA, United States). The secondary antibodies for Western blot were from Li-COR Biotechnology (Lincoln, NE, United States).

### Cell Culture

The RAW264.7 cells were from American Type Culture Collection (ATCC, Manassas, VA, United States). The cells were retained in Dulbecco’s modified Eagle’s medium (DMEM), which contained L-glutamine (2 mM), penicillin G (100 U/ml), 10% heat-inactivated FBS and streptomycin (100 mg/ml). The cells were incubated in a cell incubator remained at 37°C and a relative humidity of about 95% with 5% CO_2_. Conventional cell subculture by trypsinizing cells for 1–2 min. The cells before the 30th generation were taken for experimental research.

THP-1 human monocytic leukemia cells were from ATCC. The cells were cultured in RPMI 1640 medium containing 10% fetal bovine serum and antibiotics. THP-1 cells were treated with 50 nM PMA (Sigma-Aldrich, St. Louis, MO, United States) for 48 h to induce differentiation of the cells into macrophages. Following differentiation, non-attached cells were removed by aspiration. The adherent macrophages were then washed three times with RPMI 1640 medium and maintained in a 37°C humidified incubator containing 5% CO_2_. The cells before the 30th generation were used for the experiments.

### Cytotoxicity Assay

Cell viability was determined by MTT assay. Briefly, RAW264.7 cells were seeded in a 96-well tissue culture plate at a density of 1.4 × 10^4^ cells/well and incubated for 24 h, and then exposed to DC at various concentrations (1.25, 2.5, 5, 10, and 20 μM) for 18 h with or without LPS (100 ng/mL). THP-1 derived macrophages were seeded in a 96-well tissue culture plate at a density of 5 × 10^4^ cells/well and incubated with various concentrations (1.25, 2.5, 5, 10, and 20 μM) of DC for 24 h with or without LPS (1 μg/mL). Then, each well was added 10 μL of MTT solution (5 g/L) respectively, incubated at 37°C for 4 h, and then added 100 μL of 10% SDS-HCl solution. At last, the optical density of each well was determined at 570 nm (the reference wavelength was 650 nm).

### Measurement of NO, PGE_2_, TNF-α, and IL-6

RAW264.7 cells were seeded in a 24 well plate at a density of 8 × 10^4^ cells/well and incubated for 24 h. The cells were then pretreated with DC for 1 h, and next exposed to LPS (100 ng/mL) for 18 h. THP-1 derived macrophages (1 × 10^6^ cells/well) were seeded in 6 well plates and incubated with various concentrations (2.5, 5, and 10 μM) of DC or DEX (0.5 μM) for 1 h, finally, exposed to LPS (1 μg/mL) for 24 h.

The cell supernatant of each well was collected, respectively, for detecting the concentration of PGE_2_, TNF-α, and IL-6 with the ELISA kits according to the manufacturer’s instructions. Detection of NO content is based on Griess Reagent System (Promega, United States) according to the kit instructions.

### Real-Time PCR Analysis

RAW264.7 cells were seeded at a density of 8 × 10^4^ cells/well, and then incubated for 24 h. The cells were pretreated with various concentrations (2.5, 5, and 10 μM) of DC for 1 h, and then exposed to LPS (100 ng/mL) for 18 h. THP-1 derived macrophages (1 × 10^6^ cells/well) were seeded in 6 well plates and incubated with various concentration (2.5, 5, and 10 μM) of DC or DEX (0.5 μM) for 1 h, finally, exposed to LPS (1 μg/mL) for 24 h.

Total RNA was extracted from the cells by using the RNeasy Mini Kit (Qiagen, Germany). One microgram of total RNA was synthesized into cDNA by using the cDNA kit from Roche (Roche, Mannheim, Germany). Target RNA levels were determined by using ViiATM 7 real-time PCR.

PCR reaction system included 1 μl cDNA, 10 μl SYBR Green PCR Master Mix (Roche, Mannheim, Germany), 2 μl primers and 7 μl PCR-grade water. The reactions were performed with a denaturation step at 95°C for 10 min, 40 cycles of 95°C for 15 s and 60°C for 1 min. The relative mRNA expression levels were normalized to that of the internal control, using the 2^-ΔΔ^*^C^*^t^ cycle threshold method.

The gene relative expression for target gene were first normalized with internal reference gene (β-actin or GAPDH) and then the relative expression level of target gene for each test group were normalized with control group value. This explain why the value in the control group was 1. Therefore, the value for the normal control group was always 1 unit for each independent experiment and there is no SEM for the control group. The primers used in this study were listed in **Tables 1**, **2**, respectively.

**Table 1:** The primers for real time PCR of RAW264.7 cells.

Target gene	Primer sequences
β-actin_F	5′-CGGTTCCGATGCCCTGAGGCTCTT-3′
β-actin_R	5′-CGTCACACTTCATGATGGAATTGA-3′
iNOS_F	5′-CAGCACAGGAAATGTTTCAGC-3′
iNOS_R	5′-TAGCCAGCGTACCGGATGA-3′
COX-2_F	5′-TTTGGTCTGGTGCCTGGTC-3′
COX-2_R	5′-CTGCTGGTTTGGAATAGTTGCTC-3′
TNF-α_F	5′-TATGGCTCAGGGTCCAACTC-3′
TNF-α_R	5′-CTCCCTTTGCAGAACTCAGG-3′
IL-6_F	5′-GGTGACAACCACGGCCTTCCC-3′
IL-6_R	5′-AAGCCTCCGACTTGTGAAGTGGT-3′
Nrf2_F	5′-AGCAGGACATGGAGCAAGTT-3′
Nrf2_R	5′-TTCTTTTTCCAGCGAGGAGA-3′
HO-1_F	5′-CCCACCAAGTTCAAACAGCTC-3′
HO-1_R	5′-AGGAAGGCGGTCTTAGCCTC-3′
NQO1_F	5′-TTCTGTGGCTTCCAGGTCTT-3′
NQO1_R	5′-AGGCTGCTTGGAGCAAAATA-3′


**Table 2:** The primers for real time PCR of THP-1 cells.

Target gene	Primer sequences
GAPDH_F	5′- ACCAGCCTCAAGATCATCAGCA-3′
GAPDH_R	5′- TGCTAAGCAGTTGGTGGTGC-3′
TNF-α_F	5′- GCCCAGGCAGTCAGATCATC-3′
TNF-α_R	5′- CGGTTCAGCCACTGGAGCT-3′
IL-6_F	5′- GTGTTGCCTGCTGCCTTC-3′
IL-6_R	5′- AGTGCCTCTTTGCTGCTTTC-3′


### Protein Preparation and Western Blot Analysis

RAW264.7 cells (8 × 10^4^ cells/well) were seeded in 24 well plates. After being incubated for 24 h, the cells were treated with various concentrations (2.5, 5, and 10 μM) of DC, DEX (0.5 μM) or SFN (10 μM) for 1 h, then exposed to LPS (100 ng/mL) for 6 h or 18 h. THP-1 derived macrophages (1 × 10^6^ cells/well) were seeded in 6 well plates and incubated with various concentration (2.5, 5, and 10 μM) of DC or DEX (0.5 μM) for 1 h, finally, exposed to LPS (1 μg/mL) for 24 h.

The cells were lysed using RIPA lysis buffer and the protein concentration was detected, respectively, by using the Bradford Assay Reagent (Bio-Rad, Philadelphia, PA, United States). The nuclear and cytoplasmic protein fractions were extracted by using NE-PER nuclear and cytoplasmic extraction kit (Thermo, Pierce, United States). Equal amounts of proteins were then separated by SDS-PAGE and transferred to a nitrocellulose membrane, the membrane was blocked by using 5% BSA solution, then incubated with the corresponding primary antibody for overnight at 4°C. Then, the membranes were incubated with the secondary antibodies for 1 h at 24°C. The band of antigen-antibody complexes were scanned by using the Odyssey CLx Imager (Li-COR, United States). Western blot data were analyzed by using ImageJ software. The image densities of specific bands for target protein were first normalized with the density of loading control reference protein band (β-actin or α-tubulin). Then, the relative expression level of target protein for each test group were normalized with the control group value. This explain why the value in the control group was 1. Therefore, the value for the normal control group was always 1 unit for each independent experiment and there is no SEM for the control group.

### Immunofluorescence Analysis

For immunofluorescence analysis, RAW264.7 cells were seeded on glass coverslip in a six-well plate at a density of 2 × 10^5^ cells/well, and then incubated overnight. After being treated with 10 μM DC or SFN for 6 h, the cells were fixed with 4% paraformaldehyde for 30 min at room temperature, and subsequently permeabilized with 0.1% Triton X-100 for 30 min and blocked with 5% BSA for 30 min. The cells were then incubated with Nrf2 antibody for overnight, next incubated with the secondary antibody (Alexa Fluor 488-conjugated secondary antibody) for 1 h. At last, the cells were stained with DAPI for 5 min. The fluorescence images were captured by using a Leica TCS SP8 Confocal Laser Scanning Microscope System (Leica, Wetzlar, Germany).

### Flow Cytometric Analysis

Reactive oxygen species induction and macrophage M1 subtype were detected by flow cytometry. The cells (3 × 10^5^ cells/well) were cultured in a six-well plate, incubated for 18 h and then treated with 10 μM DC for 1 h, followed by stimulation with LPS (100 ng/mL) for 6 h. The cells were collected and stained for F/480 and CD11c to detect macrophage M1 subtype according to the manufacturer’s directions (BioLegend, San Diego, CA, United States) or stained with the fluorescent probe DCFH-DA to determine ROS level according to the manufacturer’s directions (Invitrogen, Carlsbad, CA, United States).

### Transfection Assay

Nrf2 siRNA and non-specific siRNA (NS siRNA) were transfected into RAW264.7 cells by using lipofectamine 2000 reagent according to the manufacturer’s directions. In brief, the cells were seeded in a 24-well culture plate and incubated with the NS siRNA or Nrf2 siRNA at 300 nM for 24–48 h in serum-free OPTI-MEM media (Invitrogen, United States). After incubation, the transfected cells were pretreated with indicated concentration of DC for 1 h and stimulated with or without LPS (100 ng/ml) for 18 h. The cells were prepared and the expressions of Nrf2 and HO-1 were analyzed by real-time PCR and Western blot analysis.

### Measurement of Heme Oxygenase Activity

Heme oxygenase activity was determined by the production of bilirubin. The production of bilirubin from hemin was determined upon addition of rat-liver cytosol as the source of biliverdin reductase (Srisook and Cha, 2004) with some modifications. Briefly, after the incubation, RAW264.7 macrophages were washed with PBS. Harvested cells were sonicated and centrifuged (18,000 × *g*, 10 min, 4°C). The supernatant (400 μl) was re-suspended in ice-cold potassium phosphate buffer (100 mM), then was added to a NADPH-generating system in 200 μl of reaction mixture containing 2 mg rat liver cytosol, 20 μM hemin, 1 mM NADPH, 2 mM glucose-6-phosphate, and 0.2 unit glucose-6-phosphate dehydrogenase in 100 mM potassium phosphate buffer, pH 7.4, at 37°C for 1 h. Bilirubin was then extracted with 1 ml chloroform and measured by the absorbance difference between 464 and 530 nm (extinction coefficient, 40 mM^-1^ cm^-1^ for bilirubin). HO-1 activity was expressed as nanomoles of bilirubin formed per mg of cell protein per hour.

### Statistical Analysis

The results were expressed as mean ± SEM and represented three independent experiments. Statistical analysis was carried out by using one-way ANOVA with Tukey’s multiple comparison tests or unpaired *t*-test in GraphPad Prism7. *P* < 0.05 were considered statistically significant.

## Results

### Nardochinoid C Reduced the Production of NO and PGE_2_ in LPS-Stimulated Macrophages

The MTT results showed that DC at 1.25–10 μM concentrations was non-toxic to RAW264.7 cells with or without LPS stimulation (**Figures 1B,C**). Based on these results, 2.5–10 μM DC were selected to study the anti-inflammatory activity. When the cells were exposed to LPS for 18 h, the production of NO in the cell supernatant was significantly increased (*P* < 0.01). DC obviously reduced the NO production induced by LPS in a concentration-dependent manner (*P* < 0.05, **Figure 1D**). Pretreatment with DC for 1 h also obviously and concentration-dependently reduced the production of PGE_2_ induced by LPS in LPS-stimulated RAW264.7 cells (*P* < 0.05, **Figure 1E**). DEX is a classic anti-inflammatory drug that significantly inhibits the release of inflammatory indicators (Kim et al., 2014), therefore, it was selected as a positive control to evaluate the anti-inflammatory activity of DC in this study. As shown in **Figures 1D,E**, the levels of NO and PGE_2_ were also significantly reduced by DEX (*P* < 0.01).

### Nardochinoid C Suppressed the Expression of iNOS, COX-2, TNF-α, and IL-6 in LPS-Stimulated RAW264.7 Macrophages

**FIGURE 2:**
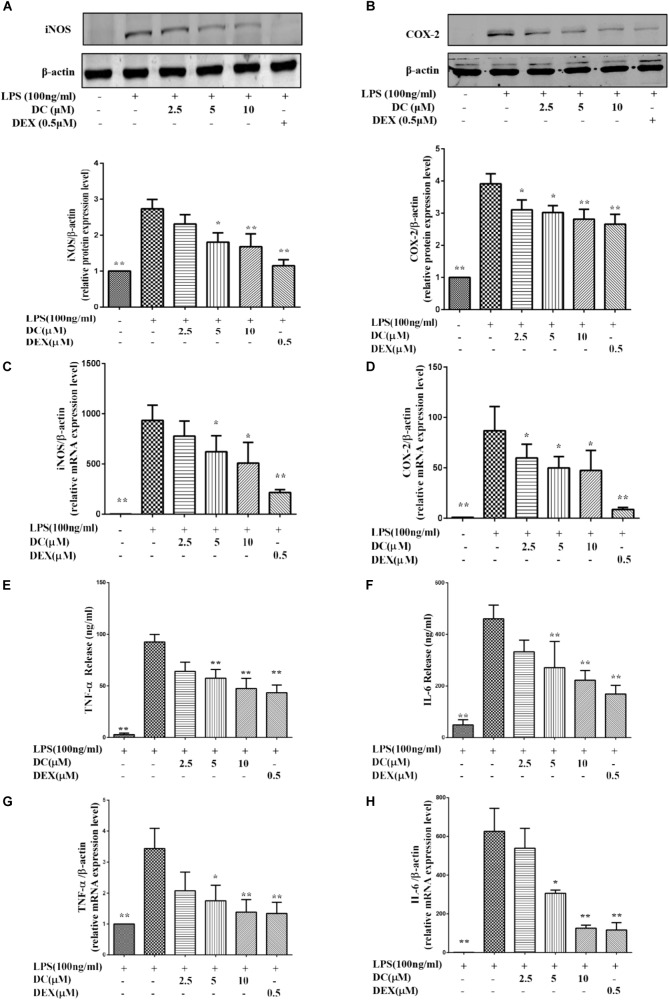
Effects of DC on the expressions of iNOS, COX-2, TNF-α, and IL-6 in LPS-stimulated RAW264.7 cells. The cells were plated in 24-well plates and incubated for 24 h, next the cells were pretreated with indicated concentrations of DC for 1 h and stimulated with LPS for 18 h. The total proteins of the cells were prepared and the expressions of iNOS **(A)** and COX-2 **(B)** were analyzed by Western blot. Total mRNA was prepared, the mRNA expressions of iNOS **(C)** and COX-2 **(D)** were analyzed by real time PCR. The protein expression levels of TNF-α **(E)** and IL-6 **(F)** in cell supernatant were analyzed by ELISA. The mRNA expression of TNF-α **(G)** and IL-6 **(H)** were analyzed by real time PCR. Statistical analysis was carried out by using one-way ANOVA with Tukey’s multiple comparison tests in GraphPad Prism7 (*P* < 0.05, ANOVA). Results are expressed as mean ± SEM of three independent experiments (*N* = 3). ^∗^*p* < 0.05, ^∗∗^*p* < 0.01, vs. LPS-stimulated cells.

In the inflammatory response, NO and PGE_2_ are synthesized by inducible nitric oxide synthase (iNOS) and cyclooxygenase-2 (COX-2), respectively (Williams et al., 1999; Sampey et al., 2005). **Figures 2A,B** showed that iNOS and COX-2 proteins were increased in LPS treatment group, while DC pre-treatment obviously and concentration-dependently decreased the elevated expression levels of iNOS and COX-2 proteins, and DEX also inhibited the expressions of iNOS and COX-2 proteins (*P* < 0.05∼0.01, **Figures 2A,B**).

As shown in **Figures 2C,D**, LPS stimulation increased the mRNA levels of iNOS and COX-2, DC pre-treatment obviously and concentration-dependently decreased the elevated mRNA levels of iNOS and COX-2, and DEX also showed inhibitory effects similar to DC (*P* < 0.05 ∼ 0.01).

When inflammation occurs, activated macrophages acts as the major effector cells that secrete large amounts of inflammatory mediators (e.g., TNF-α and IL-6) to promote the development and progression of inflammation (Fujiwara and Kobayashi, 2005). Therefore, these related inflammatory mediators, including TNF-α and IL-6 were detected in this study. As shown in **Figures 2E,F**, LPS stimulation increased the protein levels of TNF-α and IL-6, while the increased protein levels of TNF-α and IL-6 were both significantly inhibited by DC in a concentration-dependent manner and by DEX (*P* < 0.05 ∼ 0.01). LPS stimulation also increased the mRNA levels of TNF-α and IL-6 (**Figures 2G,H**), while these increases were significantly inhibited by DC concentration dependently and by DEX (*P* < 0.05 ∼ 0.01, **Figures 2G,H**).

### Nardochinoid C Suppressed the Levels of COX-2, PGE_2_, TNF-α, and IL-6 in LPS-Stimulated THP-1 Macrophages

**FIGURE 3:**
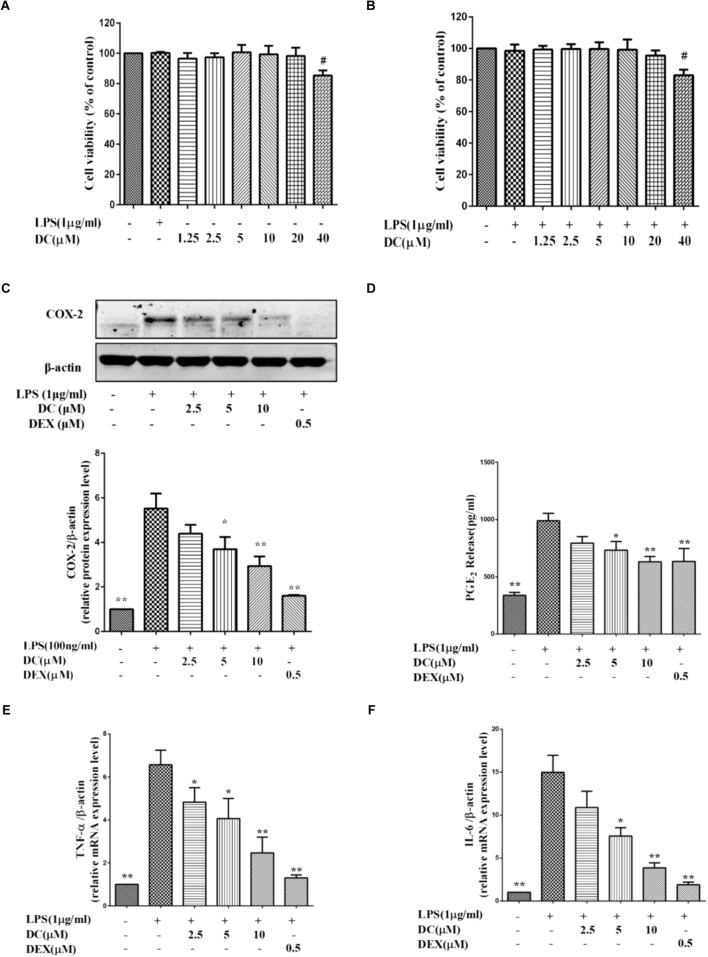
Effects of DC on the levels of COX-2, PGE_2_, TNF-α, and IL-6 in LPS-stimulated THP-1 cells. **(A)** Cytotoxicity of DC on LPS-unstimulated THP-1 cells. **(B)** Cytotoxicity of DC on LPS-stimulated THP-1 cells. **(C)** Effects of DC on the expression of COX-2 in LPS-stimulated THP-1 cells. **(D)** Effects of DC on the productions of PGE_2_ in LPS-stimulated THP-1 cells. The mRNA expression of TNF-α **(E)** and IL-6 **(F)** were analyzed by real time PCR. THP-1 cells (1 × 10^6^ cells/well) were seeded in six well plates. After being incubated for 24 h, the cells were treated with 50 nM PMA for 48 h, then incubated with various concentration (2.5, 5, and 10 μM) of DC or DEX (0.5 μM) for 1 h, finally, exposed to LPS (1 μg/mL) for 24 h. The cell supernatant, total proteins and mRNA of the cells were prepared as described before for Western blot and real time PCR. Statistical analysis was carried out by using one-way ANOVA with Tukey’s multiple comparison tests in GraphPad Prism7 (*P* < 0.05, ANOVA). Results are expressed as mean ± SEM of three independent experiments (*N* = 3). ^#^*p* < 0.05, ^##^*p* < 0.01, vs. LPS-unstimulated cells **(A,B)** or ^∗^*p* < 0.05, ^∗∗^*p* < 0.01, vs. LPS-stimulated cells **(C–F)**.

The MTT results showed that DC at 1.25–10 μM concentrations was non-toxic to THP-1 cells with or without LPS stimulation (**Figures 3A,B**). Based on these results, 2.5–10 μM DC were selected to study the anti-inflammatory activity in LPS-stimulated THP-1 macrophages.

**Figure 3C** showed that COX-2 proteins were increased in LPS treatment group, while DC pre-treatment obviously and concentration-dependently decreased the elevated expression levels of COX-2 proteins (*P* < 0.05 ∼ 0.01, **Figure 3C**).

When the cells were exposed to LPS for 24 h, the production of PGE_2_ in the cell supernatant was significantly increased (*P* < 0.01). DC obviously reduced the PGE_2_ production induced by LPS in a concentration-dependent manner (*P* < 0.05, **Figure 3D**). As shown in **Figures 3C,D**, the levels of COX-2 and PGE_2_ were also significantly reduced by DEX (*P* < 0.01).

As shown in **Figures 3E,F**, LPS stimulation also increased the mRNA levels of TNF-α and IL-6 in THP-1 cells model, while these increases were significantly inhibited by DC concentration dependently and DEX (*P* < 0.05 ∼ 0.01, **Figures 3E,F**).

### Nardochinoid C Failed to Inhibit the Activation of NF-κB and MAPK Pathway in LPS-Stimulated RAW264.7 Macrophages

Nuclear factor-κB (NF-κB) pathway is activated in inflammatory process and then promotes the expression of inflammatory mediators in different cells, including macrophages (DiDonato et al., 2012). LPS activates NF-κB pathway to induce the production of inflammatory cytokines (Ghosh and Hayden, 2008). MAPK pathway also plays an critical role in inflammatory response (Liu et al., 2007). The activation of NF-κB and MAPK signaling pathways are both involved in the development of inflammation (Liu et al., 2016). Therefore, the inhibition of NF-κB and MAPK signaling pathways are considered as the useful ways to regulate inflammatory reaction. In the non-inflammatory condition, NF-κB and IκBα are present in the cytoplasm as complex (Sahu et al., 2014). The activation of NF-κB resulted in the phosphorylation of IKKα/β, p65, and then leading to the transcription of inflammatory genes and the expression of inflammatory proteins (Ghosh et al., 1998).

**FIGURE 4:**
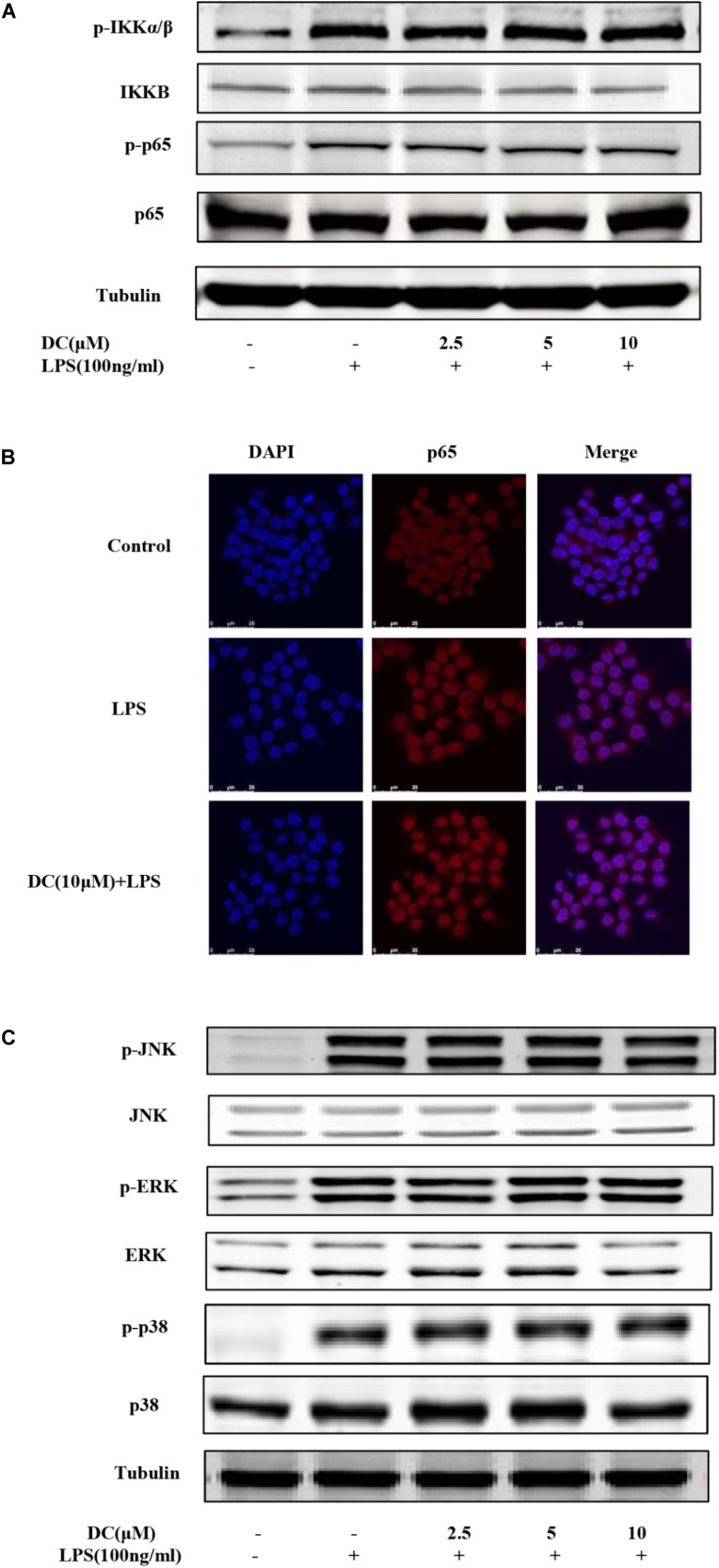
The effect of DC on the activation of NF-κB and MAPK pathways in LPS-stimulated RAW264.7 cells. The cells were plated in 24-well plates and incubated for 24 h, next the cells were pretreated with indicated concentration of DC for 1 h and stimulated with LPS for 15 min. The total proteins of the cells were prepared and the expression of phosphorylation level of IKKα/β and p65 **(A)** were detected by Western blot. The cells were treated with DC (10 μM) for 1 h and stimulated with LPS for 15 min. The subcellular localization of p65 was detected with immunofluorescence assay **(B)** and the images were acquired using the Leica DM2500 fluorescent microscopy. The total proteins of the cells were prepared and the expressions of phosphorylated JNK, ERK and p38 **(C)** were detected by Western blot.

As **Figure 4A** shows, LPS stimulation increased the phosphorylation of IKKα/β and p65. However, DC failed to inhibit the phosphorylation of these proteins (**Figure 4A**). LPS also increased the nuclear translocation of NF-κB p65 protein, but the pretreatment of DC failed to inhibit the nuclear translocation of NF-κB p65 induced by LPS (**Figure 4B**). MAPKs activation involves in regulating inflammation process (Kaminska, 2005). The activation of MAPK pathway resulted in the phosphorylation of p38, JNK and ERK (Rao, 2001), which may promote pro-inflammatory cytokines production (Sun et al., 2015). The results showed that LPS increased the protein levels of p-JNK, p-p38 and p-ERK (**Figure 4C**), but DC pretreatment didn’t inhibit the increased levels of p-JNK, p-p38 and p-ERK proteins induced by LPS (**Figure 4C**).

### Nardochinoid C Attenuated LPS-Induced M1 Phenotype and ROS Generation and Also Increased the Levels of HO-1 and NQO1 in LPS-Stimulated RAW264.7 Macrophages

**FIGURE 5:**
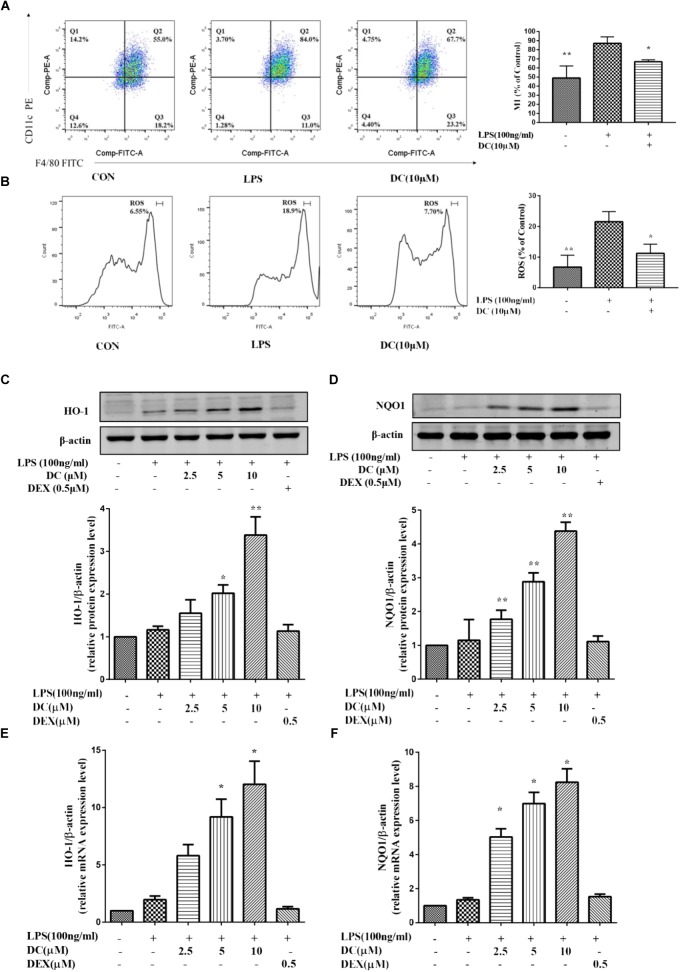
The effect of DC on the expressions of M1, ROS, HO-1, and NQO1 in LPS-stimulated RAW264.7 cells. RAW264.7 cells were pretreated with indicated concentrations of DC for 1 h, then cells were incubated for 6 h in the absence of LPS, then collected all the cells and cellular staining for F/480 and CD11c, the M1 markers **(A)** according to the manufacturer’s directions. The intracellular level of ROS **(B)** was determined using the fluorescent probe DCFH-DA. The cells were plated in 24-well plates and incubated for 24 h, next the cells were pretreated with indicated concentrations of DC for 1 h and stimulated with LPS for 18 h. The total proteins of the cells were prepared and the expression of HO-1 **(C)** and NQO1 **(D)** were analyzed by Western blot. The mRNA expressions of HO-1 **(E)** and NQO1 **(F)** were analyzed by real time PCR. Statistical analysis was carried out by using one-way ANOVA with Tukey’s multiple comparison tests in GraphPad Prism7 (*P* < 0.05, ANOVA). Results are expressed as mean ± SEM of three independent experiments (*N* = 3). ^∗^*p* < 0.05, ^∗∗^*p* < 0.01, vs. LPS-stimulated cells.

Previous reports showed that Nrf2-mediated antioxidant gene expression reduced the M1 phenotype and ROS production and contributed to anti-inflammation (Kobayashi et al., 2016). Macrophages could polarize to M1 phenotype under an inflammatory environment (Jaguin et al., 2013). As shown in **Figure 5A**, LPS induced an increase of macrophages in M1 phenotype and DC pretreatment at 10 μM suppressed this increase (*P* < 0.05). ROS participates in inflammation (Yang et al., 2015), the activation of Nrf2 could decrease the levels of ROS (Surh, 2003). As shown in **Figure 5B**, intracellular ROS was increased by LPS stimulation and DC pretreatment at 10 μM also suppressed the ROS generation induced by LPS (*P* < 0.05).

Nrf2 dimerizes with small Maf proteins in the nucleus, and then binds *cis*-oxidation reaction elements, i.e., HO-1 and NQO1, to activate their transcription (Buchman, 2001; Hotamisligil, 2006). Antioxidant protein HO-1 has anti-oxidative and anti-inflammatory effects (Linton and Fazio, 2003; McInnes and Schett, 2011). Many studies have shown that defective expression of HO-1 in humans is accompanied by an increase in the inflammatory state (Pae and Chung, 2009). Nrf2 also could increase the transcription of its target genes, including HO-1 and NQO1 (Hu et al., 2017). In order to understand whether DC could exert its anti-inflammatory effect through activating Nrf2 pathway, the downstream proteins (HO-1 and NQO1) of Nrf2 pathway were investigated in this study. As shown in **Figures 5C–F**, compared with LPS-stimulated RAW264.7 cell, DC concentration-dependently increased the levels of NQO1 and HO-1 proteins (*P* < 0.05 ∼ 0.01, **Figures 5C,D**) and the expressions of NQO1 and HO-1 mRNA (*P* < 0.05, **Figures 5E,F**). However, DEX have no obvious influence on the protein and mRNA levels of NQO1 and HO-1, compared with LPS group (**Figures 5C–F**).

### Nardochinoid C Promoted the Nucleus Translocation of Nrf2 Protein and Activated Nrf2 Pathway by Inhibiting Keap1 in LPS-Unstimulated RAW264.7 Macrophages

**FIGURE 6:**
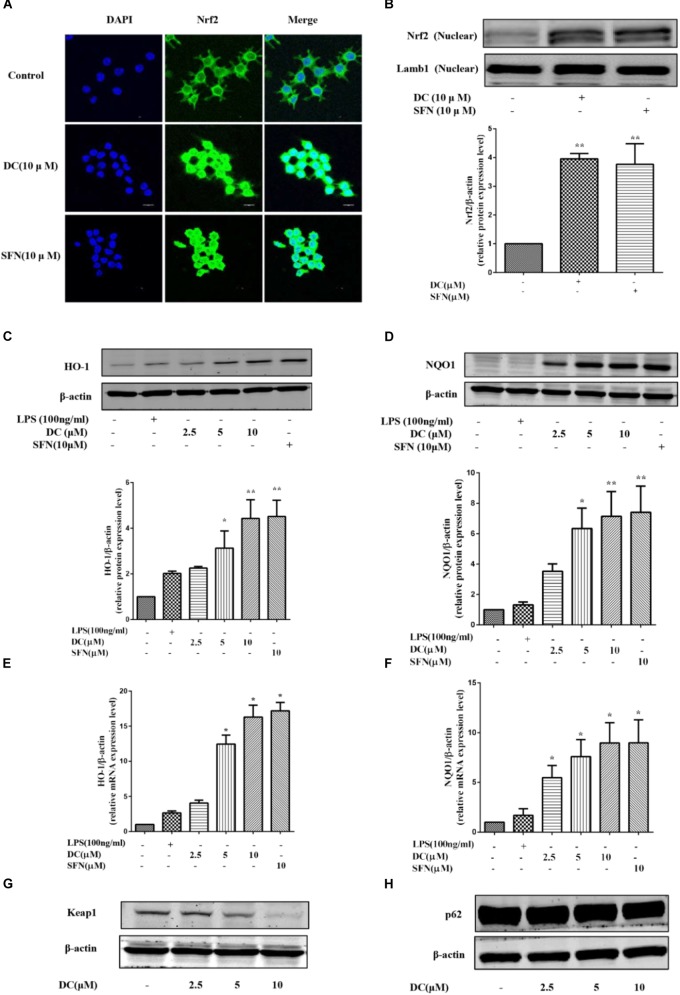
The effect of DC on the nuclear translocation of Nrf2 and the expressions of HO-1, NQO1, Keap1, and p62 in LPS-unstimulated RAW264.7 cells. The cells were treated with SFN or DC (10 μM) for 6 h. The subcellular localization of Nrf2 was detected with immunofluorescence assay **(A)** and the images were acquired using the Leica DM2500 fluorescent microscopy. The nuclear protein of the cells was prepared and the expression of Nrf2 in nuclear protein **(B)** of the cells were analyzed by Western blot. The cells were plated in 24-well plates and incubated for 24 h, next the cells were pretreated with indicated concentrations of DC for 1 h and stimulated with or without LPS for 18 h. The total proteins of the cells were prepared and the expressions of HO-1 **(C)**, NQO1 **(D)**, Keap1 **(G)**, and p62 **(H)** were analyzed by Western blot. The mRNA levels of HO-1 **(E)** and NQO1 **(F)** were analyzed by real time PCR. Statistical analysis was carried out by using one-way ANOVA with Tukey’s multiple comparison tests in GraphPad Prism7 (*P* < 0.05, ANOVA). Results are expressed as mean ± SEM of three independent experiments (*N* = 3). ^∗^*p* < 0.05, ^∗∗^*p* < 0.01, vs. LPS-unstimulated cells.

Above studies have showed that DC increased the protein levels of HO-1 and NQO1 in LPS-stimulated RAW264.7 cells. Next, the effects of DC on Nrf2 pathway in LPS-unstimulated RAW264.7 cells were also investigated. SFN, a potent Nrf2 activator (Fahey et al., 2002), was selected as a positive control. As shown in **Figures 6A,B**, pretreatment of DC at 10 μM significantly promoted Nrf2 protein entering into the nucleus in RAW264.7 cells, this is similar to SFN (*P* < 0.01). DC or SFN pretreatment both increased the level of NQO1 and HO-1 proteins (*P* < 0.05 ∼ 0.01, **Figures 6C,D**) and the expressions of NQO1 and HO-1 mRNA (*P* < 0.05, **Figures 6E,F**) in unstimulated RAW264.7 cell. Keap1 is a negative regulator of the transcription factor Nrf2 (Satoh et al., 2006). Recently, p62 was thought to be an upstream protein of Nrf2 (Komatsu et al., 2010). **Figures 6G,H** show that DC decreased Keap1 protein expression in a concentration-dependent manner but didn’t promote p62 protein expression in LPS-unstimulated RAW264.7 macrophages.

### Nrf2 siRNA and HO-1 Inhibitor Significantly Abolished the Effect of Nardochinoid C

**FIGURE 7:**
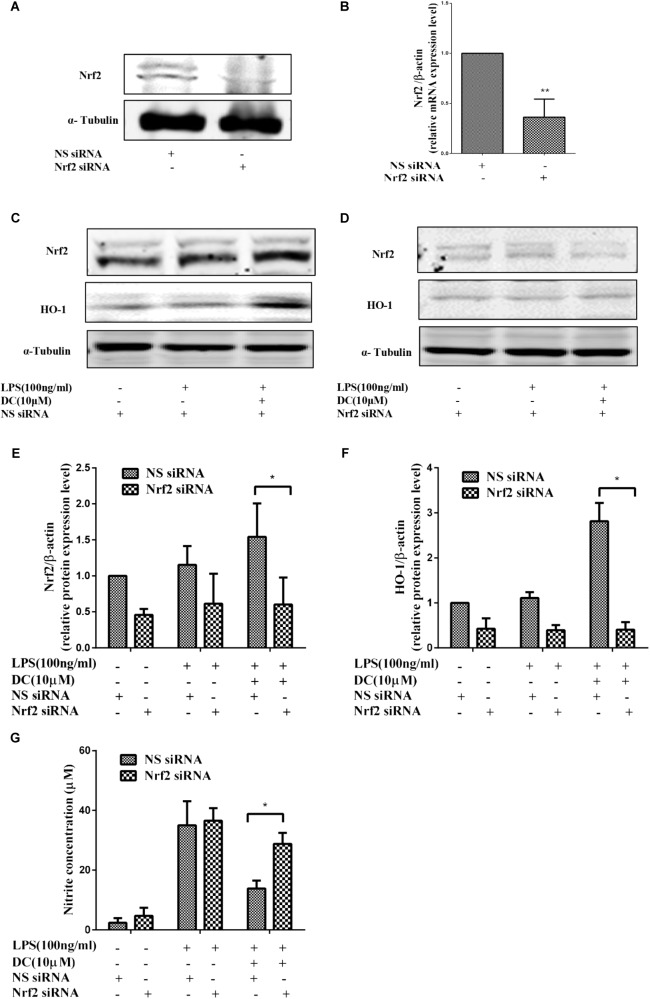
The effect of Nrf2 siRNA and HO-1 inhibitor on the anti-inflammatory effect of DC. For transfection, the cells were seeded in 24-well culture plates and incubated with the control siRNA or Nrf2 siRNA at 300 nM for 24–48 h in serum-free OPTI-MEM media. The total proteins of Nrf2 **(A)** were analyzed by Western blot. The mRNA level of Nrf2 **(B)** were analyzed by real time PCR. Statistical analysis was carried out by using unpaired *t*-test in GraphPad Prism7 (*P* < 0.05, unpaired *t*-test). Results are expressed as mean ± SEM of three independent experiments (*N* = 3). ^∗^*p* < 0.05, ^∗∗^*p* < 0.01, vs. NS siRNA treated cells **(B)**. The transfected cells were also pretreated with indicated concentration of DC for 1 h and stimulated with or without LPS for 18 h. The total proteins of the cells were prepared and the expressions of Nrf2 **(C,E)** and HO-1 **(D,F)** were analyzed by Western blot. The concentration of NO **(G)** in the culture medium were quantified. Statistical analysis was carried out by using one-way ANOVA with Tukey’s multiple comparison tests in GraphPad Prism7 (*P* < 0.05, ANOVA). Results are expressed as mean ± SEM of three independent experiments (*N* = 3). ^∗^*p* < 0.05, ^∗∗^*p* < 0.01, vs. DC, LPS, and NS siRNA treated cells **(E–G)**.

To further demonstrate the contribution of Nrf2 signaling pathway to the anti-inflammatory effect of DC, Nrf2 gene knockdown model was established by using Nrf2 siRNA transfection in RAW264.7 cells. The results showed that the protein and mRNA expressions of Nrf2 were significantly suppressed by using specific Nrf2 siRNA (**Figures 7A,B**). The increases in Nrf2 and HO-1 at protein levels caused by DC pretreatment at 10 μM were significantly suppressed by Nrf2 siRNA (**Figures 7C–F**). The suppressive effect of DC on NO production was also abolished by Nrf2 siRNA (**Figure 7G**). These results indicated that the expression of antioxidant proteins HO-1 mediated the anti-inflammatory effect of DC.

**FIGURE 8:**
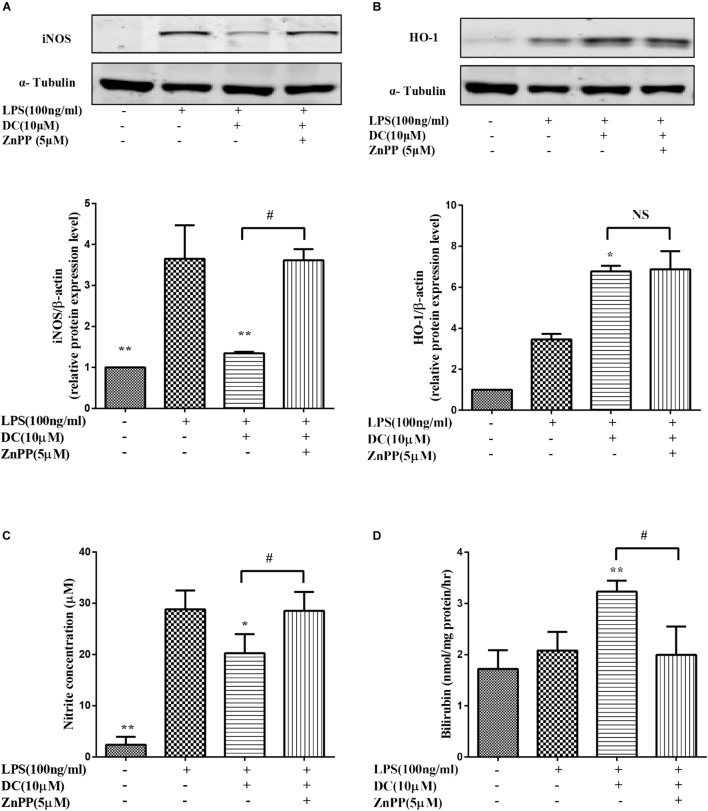
The effect of HO-1 inhibitor on the anti-inflammatory effect of DC. The cells were pretreated with indicated concentration of ZnPP for 1 h, and then stimulated with DC for 1 h, at last, treated with LPS for 18 h. The total proteins of iNOS **(A)**, and HO-1 **(B)** were analyzed by Western blot. The concentration of NO **(C)** in the culture medium were quantified. The levels of Bilirubin **(D)** in cell microsome fraction were detected. Statistical analysis was carried out by using one-way ANOVA with Tukey’s multiple comparison tests in GraphPad Prism7 (*P* < 0.05, ANOVA). Results are expressed as mean ± SEM of three independent experiments (*N* = 3). ^∗^*p* < 0.05, ^∗∗^*p* < 0.01, vs. LPS treated cells, ^#^*p* < 0.05 vs. ZnPP treated cells.

In this study, we found that DC decreased the expression of iNOS (**Figure 8A**) and the release of NO (**Figure 8C**). ZnPP, the inhibitor of HO-1 activity (Kim et al., 2008), significantly reversed the effect of DC on HO-1 protein activity (**Figure 8D**) rather than HO-1 expression (**Figure 8B**) to reverse the effect of DC on the expression of iNOS (**Figure 8A**) and the release of NO (**Figure 8B**).

## Discussion

When inflammation occurs, activated macrophages release a large number of different inflammatory mediators (NO, PGE_2_, TNF-α, and IL-6) and regulatory enzymes (iNOS and COX-2) (Linton and Fazio, 2003; Chawla et al., 2011; McInnes and Schett, 2011; Balkwill and Mantovani, 2012). In this research, LPS stimulation elevated the levels of these inflammatory mediators (NO, PGE_2_, TNF-α, and IL-6) and regulatory enzymes (iNOS and COX-2), but the increases of these inflammatory mediators and regulatory enzymes were significantly inhibited by DC (**Figures 1**, **2**), implying that DC has obvious anti-inflammatory activity in LPS-stimulated RAW264.7 macrophage cells model.

THP-1 cell line was used to estimate modulation of monocyte and macrophage activities (Chanput et al., 2014). In this study, we also use LPS-stimulated THP-1 macrophages to study the anti-inflammatory activity of DC. Our results showed that LPS stimulation elevated the levels of inflammatory protein (COX-2), inflammatory mediators (PGE_2_) and inflammatory genes (TNF-α and IL-6), but these increases were significantly inhibited by DC (**Figures 3D,E**), implying that DC also has obvious anti-inflammatory activity in LPS-stimulated THP-1 macrophages, these effects are similar to these in LPS-stimulated RAW264.7 macrophages.

In LPS-stimulated THP-1 cell model, we did not detect the high expressed iNOS gene (data not shown), which is similar to the paper reported that LPS could not up-regulated iNOS gene in THP-1 macrophages (Chanput et al., 2010). There is no doubt that iNOS is an important inflammatory index, it is expressed in response to a variety of inflammatory stimuli and generates NO in macrophage during the inflammatory process (Laskin and Pendino, 1995), the inhibition of iNOS was able to reduce the production of ROS (Egea et al., 2012). As described earlier, ROS play an important role in oxidative stress and inflammatory responses. LPS-stimulated THP-1 macrophages without the high expressed iNOS gene, so in the next study, we selected the commonly used inflammatory model, LPS-stimulated RAW264.7 macrophages which expresses high levels of iNOS and ROS for in-depth study on the mechanisms underlying the anti-inflammatory effect of DC.

There were reports showing that the inhibition of LPS-induced NO, PGE_2_, iNOS, COX-2, TNF-α, and IL-6 through the inactivation of NF-κB and MAPK pathway in RAW264.7 cells (Kim et al., 2007; Liu et al., 2016). Other previous studies have introduced that the extracts of *N. chinensis* inhibited the production of inflammatory mediators through the inhibition of p38 MAPK pathway instead of the inhibition of NF-κB pathway. In order to study the anti-inflammatory mechanism of DC, the effects of DC on the activation of NF-κB and MAPK pathways in LPS-stimulated RAW264.7 cells were investigated, but the results suggested that DC didn’t inhibit the activation of NF-κB and MAPK pathways (**Figures 4A–C**), so DC may not act on NF-κB and MAPK pathways to exert its anti-inflammatory effects. This is common to other terpenoid- derived compounds with known anti-inflammatory effects (Ferrante et al., 2017; Locatelli et al., 2017).

It has been reported that the activation of Nrf2 anti-oxidant pathway prevents LPS-induced transcriptional upregulation of pro-inflammatory cytokines, including IL-6. Since the current results showed that DC inhibited IL-6 obviously (**Figure 2F**), so it was hypothesized that DC may activate Nrf2 pathway to exert its anti-inflammatory effect. As described before, Nrf2 pathway mediated antioxidant gene expression reduced the M1 phenotype and ROS production (Kobayashi et al., 2016). In order to verify the hypothesis of this study, the M1 phenotype and ROS production in LPS-stimulated RAW264.7 cells were examined, the results showed that the elevated M1 phenotype and ROS production induced by LPS were significantly inhibited by DC (**Figures 5A,B**), suggesting that DC is likely to exert anti-inflammatory and anti-oxidant effects by activating the Nrf2 anti-oxidant pathway.

The Nrf2-dependent anti-oxidant genes HO-1 and NQO-1 could block TNF-α and IL-6 inflammatory mediators. In Nrf2-knockout mice, the anti-inflammatory effect disappeared (Thimmulappa et al., 2006). Nrf2-knockout mice showed increased mRNA and protein levels of COX-2, iNOS, IL-6, and TNF-α (Rojo et al., 2010). Furthermore, the activation of Nrf2 leads to its nuclear translocation, resulting in the decrease of COX-2 and iNOS (Ho et al., 2007). In this study, it was found that DC inhibited the expressions of inflammatory proteins (COX-2 and iNOS, **Figures 2A,B**) and inflammatory cytokines (TNF-α and IL-6, **Figures 2E–H**) accompanied by the increase of antioxidant proteins HO-1 and NQO1 (**Figures 5C–F**). All of these results further indicated that DC may activate Nrf2 pathway to exert its anti-inflammatory effects.

In oxidative stress and inflammation condition, enhancement of HO-1 expression plays an important role in cell protection (Ryter et al., 2006; Chung et al., 2008). HO-1 can be rapidly induced by various oxidative-inducing agents, also including LPS (Chung et al., 2008). The current results also showed that LPS increased the level of HO-1 slightly, but compared with LPS group, DC further increased the level of HO-1 protein dramatically (**Figures 5C,E**). The high expression of HO-1 can inhibit LPS-induced NO production (Lin et al., 2003). In this study, DC reduced NO production while increasing HO-1 expression (**Figures 1D**, **5C,E**), the current results are consistent with that the high expression of HO-1 can inhibit LPS-induced NO production (Lin et al., 2003).

Sulforaphane is an known and potent Nrf2 activator and capable of preventing toxicity of organic chemicals (Fahey et al., 2002) and has antioxidant protection effects (Gao et al., 2001). In the current study, DEX had strong anti-inflammatory activity, but didn’t show any effect on the activation of Nrf2 pathway, implying that DEX produced its anti-inflammatory effect through a Nrf2 independent way. Therefore, SFN was chosen as a positive drug to evaluate the antioxidant activity and underlying mechanisms of DC related to Nrf2 pathway. The current research showed that both DC and SFN increased the levels of HO-1 and NQO1 in LPS-unstimulated RAW264.7 cell (**Figures 6C–F**). Since DC increased the downstream antioxidant proteins (HO-1 and NQO1) of Nrf2 pathway, it was indicated that DC could also activate Nrf2 and make Nrf2 protein entering into the nucleus. In this research, we did find that DC increased the nuclear translocation of Nrf2 (**Figures 6A,B**), further supported that DC was able to activate Nrf2 pathway to promote the increase of antioxidant protein to reduce ROS production.

Keap1 is a negative regulator of the transcription factor Nrf2, which mediated the inactivation of Nrf2 and thus enhanced Nrf2 translocation into the nucleus (Satoh et al., 2006; Lv et al., 2018). It has been reported that protein p62, which is on the upstream of Nrf2 pathway, can inactivate Keap1 and activate Nrf2 (Komatsu et al., 2010). The current research found that DC inhibited the expression of Keap1 (**Figure 6G**) but didn’t increase p62 expression (**Figure 6H**), suggesting that DC induced Nrf2 activation probably through inhibiting Keap1 expression instead of upregulating p62 protein.

To further confirm the pivotal role of Nrf2 pathway in the anti-inflammatory effect of DC, Nrf2 siRNA was employed to down regulate the protein and gene expressions. The results showed that the mRNA and protein level of Nrf2 were significantly suppressed by using specific Nrf2 siRNA (**Figures 7A,B**). The protein expressions of Nrf2 and HO-1 were decreased by Nrf2 siRNA (**Figures 7C–F**). The suppressive effect of DC on NO production induced by LPS was also abolished by Nrf2 siRNA (**Figure 7G**), these results suggested the anti-inflammatory effect of DC is mediated by the activation of Nrf2/HO-1 antioxidant pathway.

It has been reported in the literature (Srisook et al., 2006) that increasing the activity of HO-1 can reduce the production of iNOS and NO. Bilirubin, the metabolic products of HO-1, also exerts anti-inflammatory effect (Araujo et al., 2012). In order to further confirm the important role of HO-1 activity in mediating the anti-inflammatory effect of DC. ZnPP, a HO-1 activity inhibitor (Freitas et al., 2006), was used in the research.

**FIGURE 9:**
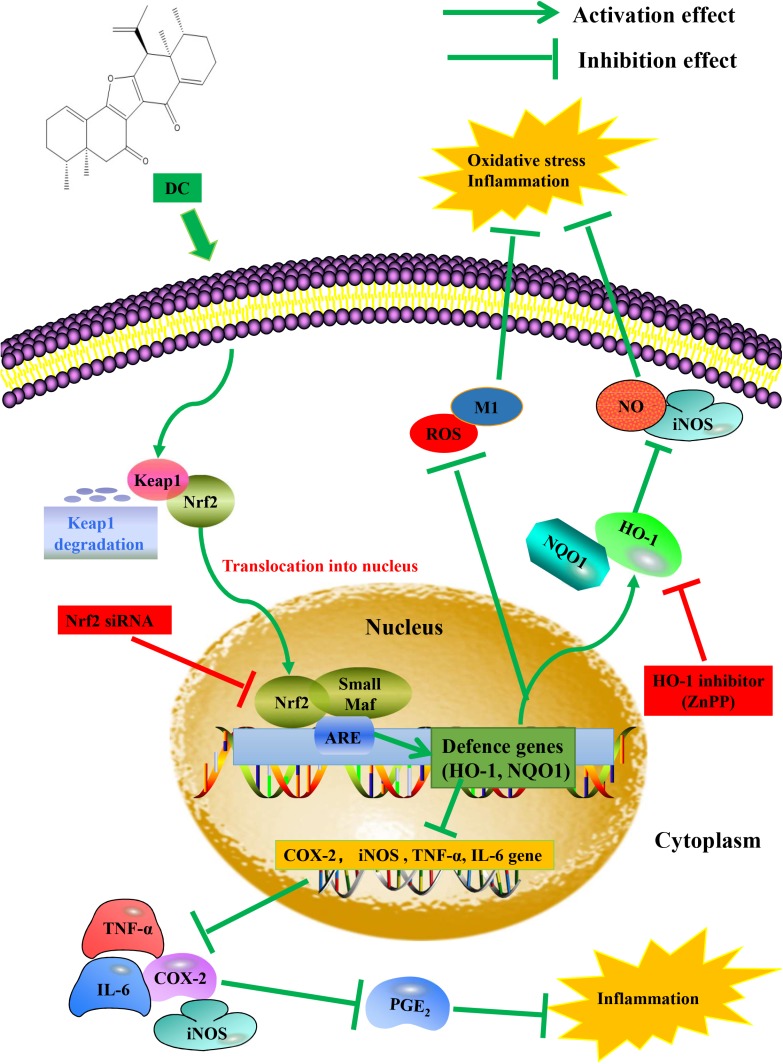
Proposed molecular mechanisms underlying the inhibitory effect of DC on the activation of macrophage induced by LPS. DC activates Nrf2 pathway to increase anti-oxidant genes and protein, which in turn reduce inflammation and oxidative stress.

In this study, we found that DC increased the expression (**Figure 8B**) and the activity of HO-1 protein (**Figure 8D**). At the same time, DC decreased the expression of iNOS (**Figure 8A**) and the release of NO (**Figure 8C**). ZnPP significantly reversed the effect of DC on HO-1 protein activity (**Figure 8D**) rather than HO-1 expression (**Figure 8B**) to block the effect of DC on the expression of iNOS (**Figure 8A**) and the release of NO (**Figure 8C**), these results further confirmed that the anti-inflammatory effect of DC was mediated by the increase of HO-1 activity. In short, DC activates Nrf2/HO-1 pathway to increase anti-oxidant proteins, which in turn reduce inflammation and oxidative stress to contribute to its anti-inflammatory and antioxidant effects (**Figure 9**).

Nrf2 activator can be used as potential therapies for numerous disorders (Crunkhorn, 2012), but there are very few Nrf2 activators applicable currently in clinics. In addition, the current available Nrf2 activator, dimethyl fumarate, has showed drug resistance and side effects (Deeks, 2014). Therefore, there is significant need to develop new and safer Nrf2 activator for clinical use. In this research, DC exerted anti-inflammatory effects mainly through the activation of Nrf2 pathway. This implies that DC may be a new Nrf2 activator and has the potential to be developed as new drug. Nrf2 pathway plays an important role in oxidative stress response and metabolism, Nrf2 activation can significantly inhibit the onset of diabetes and prevent diabetes (Uruno et al., 2013). Since DC also activates Nrf2 pathway in the absence of LPS, it indicated that DC could not only treat inflammation but also prevent many other diseases related to oxidative stress response and metabolism, giving broader application situations to this compound.

## Conclusion

Taken together, we discovered a novel Nrf2 activator, DC, which acts on Nrf2/HO-1 pathway to exert its anti-inflammatory effects against LPS-induced inflammation. Besides, the unique anti-inflammatory mechanism of DC may provide a new therapeutic window for the prevention and treatment of oxidative damage and inflammation-related diseases. Although other inflammation-related cell experiments, animal models and clinical trials are certainly needed to do in future depth research, this study now still could help providing a potential treatment mechanism of *N. chinensis* and also provide a novel natural compound with new skeleton for the treatment of the diseases related to inflammation and oxidative stress.

## Author Contributions

J-FL performed the experiments, analyzed the data, and wrote the paper. X-YS and YD provided the compound. CKL performed the experiments. C-SC, J-XL, and Y-DY analyzed the data. YY, YX, PL, and X-SY critically read the manuscript. Z-QL and HZ conceived the research idea and revise the manuscript. All authors contributed to final approval of the article.

## Conflict of Interest Statement

The authors declare that the research was conducted in the absence of any commercial or financial relationships that could be construed as a potential conflict of interest.

## Abbreviations

AD: Alzheimer’s disease
BSA: bovine serum albumin
COX-2: cyclooxygenase-2
DC: nardochinoid C
DCFH-DA: 2,7-dichlorodihydrofluorescein diacetate
DEX: dexamethasone
HO-1: heme oxygenase-1
IL-6: interleukin-6
iNOS: nitric oxide synthase
Keap1: Kelch-like ECH-associated protein 1
LPS: lipopolysaccharide
Maf: musculoaponeurotic fibrosarcoma
MAPK: mitogen-activated protein kinases
NF-κB: nuclear factor-κB
NO: nitric oxide
NQO1: NAD(P)H quinoneoxidoreductase 1
Nrf2: nuclear factor-E2-related factor 2
PGE_2_: prostaglandin E_2_
PMA: phorbol myristate acetate
RA: rheumatoid arthritis
ROS: reactive oxygen species
SFN: sulforaphane
siRNA: small interfering RNA
TNF-α: tumor necrosis factor-α
ZnPP: zinc protoporphyrin IX
